# Human α_2_β_1_^HI^ CD133^+VE^ Epithelial Prostate Stem Cells Express Low Levels of Active Androgen Receptor

**DOI:** 10.1371/journal.pone.0048944

**Published:** 2012-11-07

**Authors:** Stuart C. Williamson, Anastasia C. Hepburn, Laura Wilson, Kelly Coffey, Claudia A. Ryan-Munden, Deepali Pal, Hing Y. Leung, Craig N. Robson, Rakesh Heer

**Affiliations:** 1 Northern Institute for Cancer Research, Newcastle University, Newcastle upon Tyne, Tyne and Wear, United Kingdom; 2 The Beatson Institute for Cancer Research, Glasgow, East Dunbartonshire, United Kingdom; University of Cincinnati, United States of America

## Abstract

Stem cells are thought to be the cell of origin in malignant transformation in many tissues, but their role in human prostate carcinogenesis continues to be debated. One of the conflicts with this model is that cancer stem cells have been described to lack androgen receptor (AR) expression, which is of established importance in prostate cancer initiation and progression. We re-examined the expression patterns of AR within adult prostate epithelial differentiation using an optimised sensitive and specific approach examining transcript, protein and AR regulated gene expression. Highly enriched populations were isolated consisting of stem (α_2_β_1_^HI^ CD133^+VE^), transiently amplifying (α_2_β_1_^HI^ CD133^–VE^) and terminally differentiated (α_2_β_1_^LOW^ CD133^–VE^) cells. AR transcript and protein expression was confirmed in α_2_β_1_^HI^ CD133^+VE^ and CD133^–VE^ progenitor cells. Flow cytometry confirmed that median (±SD) fraction of cells expressing AR were 77% (±6%) in α_2_β_1_^HI^ CD133^+VE^ stem cells and 68% (±12%) in α_2_β_1_^HI^ CD133^–VE^ transiently amplifying cells. However, 3-fold lower levels of total AR protein expression (peak and median immunofluorescence) were present in α_2_β_1_^HI^ CD133^+VE^ stem cells compared with differentiated cells. This finding was confirmed with dual immunostaining of prostate sections for AR and CD133, which again demonstrated low levels of AR within basal CD133^+VE^ cells. Activity of the AR was confirmed in prostate progenitor cells by the expression of low levels of the AR regulated genes PSA, KLK2 and TMPRSS2. The confirmation of AR expression in prostate progenitor cells allows integration of the cancer stem cell theory with the established models of prostate cancer initiation based on a functional AR. Further study of specific AR functions in prostate stem and differentiated cells may highlight novel mechanisms of prostate homeostasis and insights into tumourigenesis.

## Introduction

Androgen signalling has been shown to be integral to prostate cancer development as it can induce and regulate *TMPRSS2*–*ERG* gene fusions, which initiate malignant transformation and drive disease progression [1]–[3]. Even without this fusion, AR signalling remains central to prostate carcinogenesis [4]–[6].

There is increasing evidence that stem cells are the targets for tumourigenesis due to their inherent self-renewal capability, anti-apoptotic pathways and maintenance throughout the lifetime of an individual granting time for mutations to accumulate. Human studies of tumourigenesis in xenografts have demonstrated the importance of AR signalling in disease initiation in the basal layer of prostate epithelium [6]. In mice, evidence is growing that there are both basal and luminal stem cells and debate remains over where the critical tumourigenic mutations occur, nevertheless both these models of carcinogenesis required an active AR [7]–[11]. In the human setting, a common clonal origin has been confirmed for basal, luminal and neuroendocrine cells [12], [13]. Human prostate stem cells can be enriched by their gene signature of α_2_β_1_^HI^ and glycosylated CD133 expression, transiently amplifying cells are characterised by α_2_β_1_^HI^ CD133^–VE^ expression and terminally differentiated cells are defined by the marker α_2_β_1_^LOW^ CD133^–VE^ [14]–[17]. Both stem cells and cancer stem cells described by these signatures from primary human prostates have typically lacked AR expression [14], [18]. The existence of AR^–VE^ cancer stem cells has been postulated as a mechanism by which tumours relapse by overcoming androgen ablative therapies that target AR^+VE^ cells [18]. However, it is established that the AR remains active and even amplified in castration resistant prostate cancer (CRPC) [19]–[21]. If the prostate stem cell is the cell of origin for transformation, then this model appears to be at odds with the emerging mechanisms of prostate cancer development and progression dependent upon AR signalling. In this work, we focus on re-examining the expression profiles of AR in prostate epithelial differentiation and challenge the dogma that prostate stem cells lack AR.

## Methods

### Tissue Collection and Isolation of Epithelial Cells

Human prostate samples were obtained from 20 patients following transurethral resection of the prostate for benign prostatic hyperplasia or cystoprostatectomy for bladder cancer. Pathologist assessment confirmed benign histology and the samples underwent processing and selection as previously described [14]–[16]: Magnetic activated cell sorting (MACS) was performed for immunomagnetic selection of Epithelial Cell Adhesion Molecule (EpCAM/CD326) (Miltenyi Biotec, Woking, UK). Epithelial α_2_β_1_^HI^ (stem and transiently amplifying cells) and α_2_β_1_^LOW^ (differentiated) cells were selected by rapid adhesion to collagen-1. Epithelial α_2_β_1_^HI^ CD133^+VE^ cells were separated by either CD133 immunomagnetic selection (CD133/1, Miltenyi Biotec) or FACS (CD133/2, Miltenyi Biotec). In our work, selected primary samples were never cultured prior to experimentation to avoid adaptations of cells in an *in vitro* environment and subsequent deviation of their phenotypes [22]–[24].

### Maintenance of Prostate Cancer Cell Lines

The human prostate cancer cell lines LNCaP (AR^+VE^) and PC3 (AR^–VE^) (American Type Culture Collection) were maintained in RPMI1640 medium (Sigma, Dorset, UK) containing 10% foetal calf serum and 2 mM L-glutamine.

### siRNA Knockdown of AR

Cells were seeded in six well plates prior to being transfected with either small interfering RNA (siRNA) (Sense strand 5′ CCAUCUUUCUGAAUGUCCU dTdT 3′, antisense 5′ AGGACAUUCAGAAAGAUGG dTdT 3′) for AR or non-silencing siRNA (Sense strand 5′ UUCUCCGAACGUGUCACGU dTdT 3′, antisense 5′ ACGUGACACGUUCGGAGAA dTdT 3′) using Lipofectamine™ RNAiMAX (Invitrogen, Paisley, UK).

### Quantitative Real Time PCR Analysis

Prostate epithelia was separated into three distinct fractions, α_2_β_1_^HI^ CD133^+VE^ stem cells, α_2_β_1_^HI^ CD133^–VE^ transiently amplifying cells and α_2_β_1_^LOW^ CD133^–VE^ terminally differentiated cells and underwent RNA isolation (micro RNeasy, Qiagen, Crawley, UK). Message BOOSTER™ cDNA synthesis amplification kit (Epicentre Biotechnologies, Madison, WI, USA) was employed and real-time PCR (Applied Biosystems 7900HT) was performed using SYBR green (Invitrogen) using the following specific primer sets: AR (forward 5′-CTG GAC ACG ACA ACA ACC AG-3′, reverse 5′-CAG ATC AGG GGC GAA GTA GA-3′), PSA (forward 5′-CAA TGA CGT GTG TGC GCA A-3′, reverse 5′-CGT GAT ACC TTG AAG CAC ACC A-3′), KLK2 (forward 5′-AGC ATC GAA CCA GAG GAG TTC T-3′, reverse 5′-TGG AGG CTC ACA CAC TGA AGA-3′), TMPRSS2 (forward 5′-CTG CTG GAT TTC CGG GTG-3′, reverse 5′-TTC TGA GGT CTT CCC TTT CTC CT-3′), CD24 (forward 5′-TGA AGA ACA TGT GAG AGG TTT GAC-3′, reverse 5′-GAA AAC TGA ATC TCC ATT CCA CAA-3′), CD146 (forward 5′-CCA TTT TTG GCC CCC CT-3′, reverse 5′TCA CCC ACA CCT TCC TCT CCT-3′) and CD45 (forward 5′-GAA ATT GTT CCT CGT CTG AT-3′, reverse 5′-CTT TGC CCT GTC ACA AAT AC-3′) before being normalised to GAPDH (forward 5′-CGA CCA CTT TGT CAA GCT CA-3′, reverse 5′-GGG TCT TCC TTG GAG GC-3′).

### Flow Cytometry

Cells were fixed with Fixation/Permeabilisation solution (BD Bioscience, Oxford, UK) before incubation in methanol at −20°C for 16 hours to permeabilise the nucleus. Cells were labelled with anti-AR antibody (PG-21, Millipore) and secondary FITC (Dako, Ely, UK), and CD133/2 antibody directly conjugated to PE (Miltenyi Biotec). Controls included IgG isotype antibody (Dako) and PE conjugated isotype antibody (Miltenyi Biotec). When required, cells were counterstained with the nuclear stain DRAQ5™ (Biostatus, Shepshed, UK) according to the manufacturer’s recommendations. Samples were analysed using either a FACS Calibur flow cytometer (BD Biosciences) or ImageStreamX Mark II cytometer (Amnis, Ipswich, UK).

### Western Blot Analysis

Resolved lysates (12% polyacrylamide gel) were transferred to Hybond C membranes (Amersham Biosciences, Amersham, UK) and probed with either 1∶1000 AR (G122-434, BD Pharmingen), or 1∶4000 α-tubulin (T9026, Sigma) antibodies, followed by 1∶500 rabbit anti-mouse HRP secondary antibody (DAKO) for subsequent visualization using the ECL detection system (GE Healthcare, Little Chalfont, UK).

### Sequential Alkaline Phosphatase Immunostaining

CD133/AR dual staining was carried out on prostate sections using methodologies previously described [25]. Briefly, sections were stained with CD133/1 antibody (Miltenyi Biotec) according to manufacturer’s recommendations prior to detection using Poly-AP-GAM/R/R Immunoglobulins (Immunologic, Duiven, The Netherlands) and visualised with Alkaline phosphatase substrate kit III (SK-5300, Vector labs, Burligame, Ca, USA). Sections were cleared of antibodies with a second antigen retrieval before staining for AR (SC-816, Santa Cruz), detection with Poly-AP-GAM/R/R Immunoglobulins and visualisation with Alkaline phosphatase substrate kit I (SK-5100, Vector labs) and mounting slides in Vecta Mount (H-5000, Vector labs).

## Results

### The Purity of Human EpCaM^+VE^ α_2_β_1_^HI^ CD133^+VE^ Prostate Cell Selections was Confirmed

**Figure 1:**
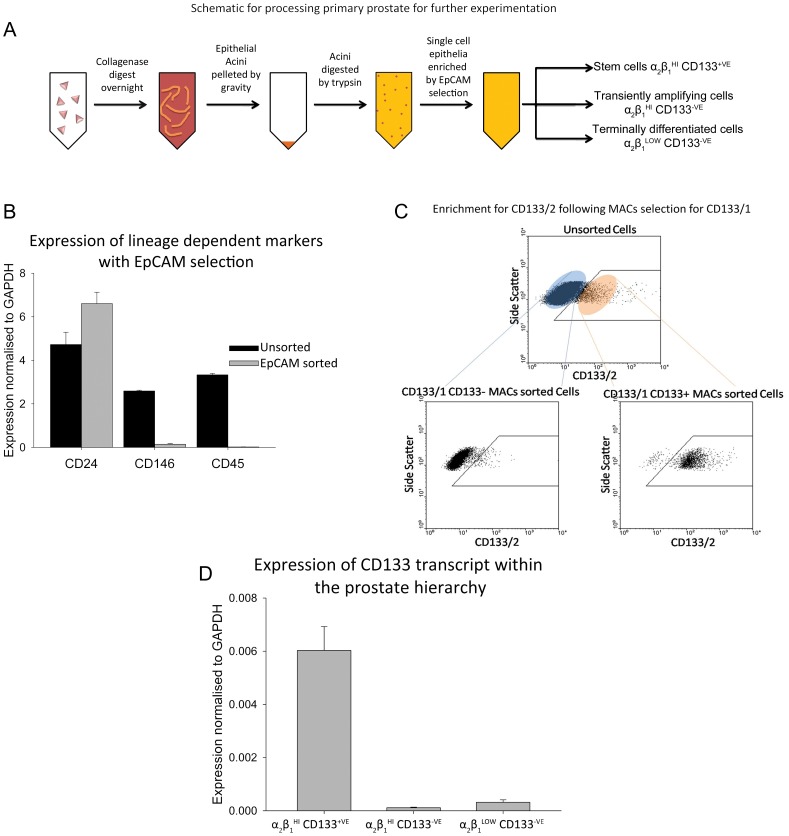
Strategy of enrichment for required cell types. **A)** Schematic work flow for enrichment of epithelial cells for assessment of AR expression. **B)** Purity of selection by expression of the lineage specific markers CD24 (epithelial), CD146 (endothelial) and CD45 (haematopoietic) normalised to GAPDH following real-time PCR for unsorted prostate epithelia and EpCAM/HEA sorted epithelia, error bars represent standard error of the mean for n = 3. **C)** CD133/1 Sorted samples were assessed for purity by co-expression of the CD133/2 epitope, confirming that these two epitopes are co-expressed in the prostate and that our CD133 selection efficiently enriches for CD133^+VE^ cells: Upper dotplot representative of CD133/2 staining for unsorted α_2_β_1_^HI^ epithelial cells; lower left dotplot representative of CD133/2 staining for α_2_β_1_^HI^ CD133/1^–VE^ cells; lower right dotplot representative of CD133/2 staining for α_2_β_1_^HI^ CD133/1^+VE^ cells. Gates are set according to appropriate isotype controls. **D)** Confirmation of CD133 enrichment with real-time PCR. CD133 expression is shown normalised to GAPDH, error bars represent standard error of the mean n = 10.

It is established that primary culture results in changes in phenotype from those seen *in vivo* [23], [24], [26] and of particular relevance, studies in glioma have demonstrated that CD133 expression is altered as a result of *in vitro* conditions [27]. Therefore, in our studies no culturing of samples was carried out prior to experimentation. However, this approach can lead to a greater chance of contamination by unwanted cell lineages, such as blood or stroma cell types, and a previously optimised protocol for epithelial extraction was employed (Figure 1A) [14]–[16], [28], [29]. Lineage-specific markers for epithelial (CD24), endothelial (CD146) and haematopoietic (CD45) cells were assessed and the purity of epithelial cell enrichment was confirmed by real time PCR, confirming depletion of unwanted cell lineages with this method (Figure 1B). In order to assess the enrichment of glycosylated CD133, cells were dual-stained for CD133/1 immunomagnetic beads and an anti-CD133/2 antibody that targets an epitope distinct from CD133/1. Flow cytometry data showed that in the prostate CD133/1 and CD133/2 are exclusively co-expressed, confirming >98% purity in glycosylated-CD133^+VE^ cells (Figure 1C). Similarly using real time PCR, immunomagnetic selection for glycosylated-CD133 resulted in enrichment for cells expressing high levels of CD133 mRNA and depleted CD133 expression in the negative fractions (Figure 1D).

### The Sensitivity and Specificity of AR Detection by Flow Cytometry was Validated

**Figure 2:**
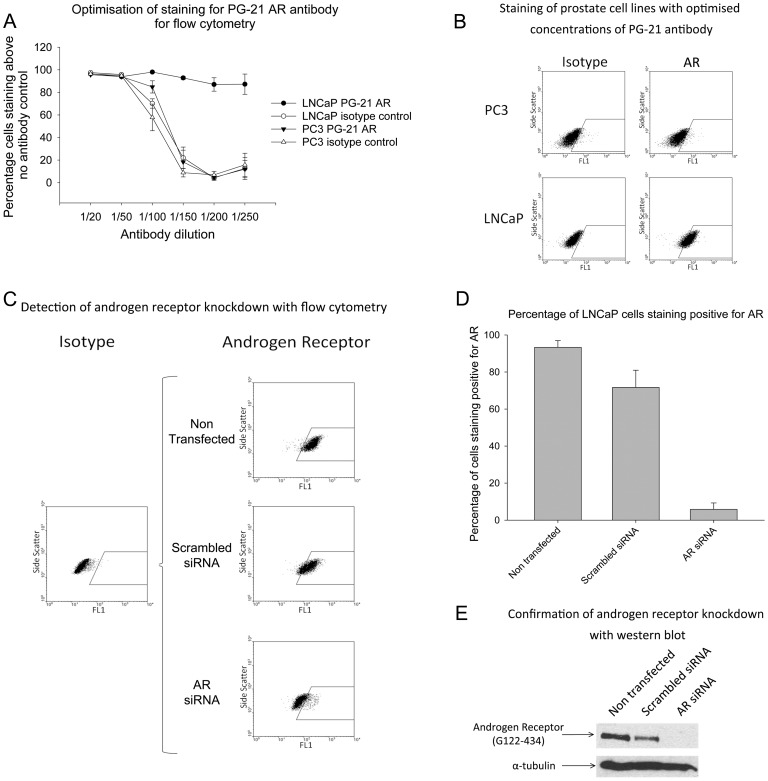
Validation of AR detection with flow cytometry. **A)** Percentage of cells staining above a no antibody control for either isotype antibody (hollow points) or PG-21 AR antibody (solid points) in LNCaP (circles) or PC3 (triangles) across a dilution series. **B)** Representative staining patterns for PC3 (upper dotplots) and LNCaP (lower dotplots) for either 1∶200 isotype antibody (left dotplots) or 1∶200 PG-21 AR antibody (right dotplots) of equivalent concentrations. Gates set according to isotype control. **C)** Left dotplot representative of staining of LNCaP with isotype control, right dotplots representative of AR staining in non-transfected LNCaP (upper), LNCaP transfected with scrambled siRNA (middle dotplot) and LNCaP transfected with AR siRNA (lower). Gates were set according to an appropriate isotype control. **D)** Percentage of cells staining positive for AR relative to an isotype control in non-transfected LNCaP, LNCaP transfected with scrambled siRNA and LNCaP transfected with AR siRNA. Error bars represent standard error of the mean for n = 3. **E)** Western blots of AR expression for non-transfected LNCaP, LNCaP transfected with scrambled siRNA and LNCaP transfected with AR siRNA are shown using a different AR antibody (G122-434, BD Pharmingen).

In order to accurately determine the presence of the AR in rare cell types within the prostate, a flow cytometry approach was developed, allowing both sensitive and specific quantification. Using a specific AR antibody (PG-21, Millipore), an optimised staining protocol was developed using prostate cancer cell lines LNCaP (AR^+VE^) and PC3 (AR^–VE^), allowing identification of the highest concentrations of antibody to increase sensitivity whilst controlling for non-specific labelling (Figure 2A). Specific AR expression was confirmed by comparison to an IgG-specific isotype control in conjunction with the AR^–VE^ cell line PC3 (Figure 2B). Specificity of this staining was further confirmed in LNCaP following siRNA knockdown of the AR using flow cytometry (Figure 2C and 2D). Western Blot analysis was also employed to confirm the specificity of the AR knockdown with siRNA, which correlated directly with the flow cytometry result (Figure 2E).

**Figure 3:**
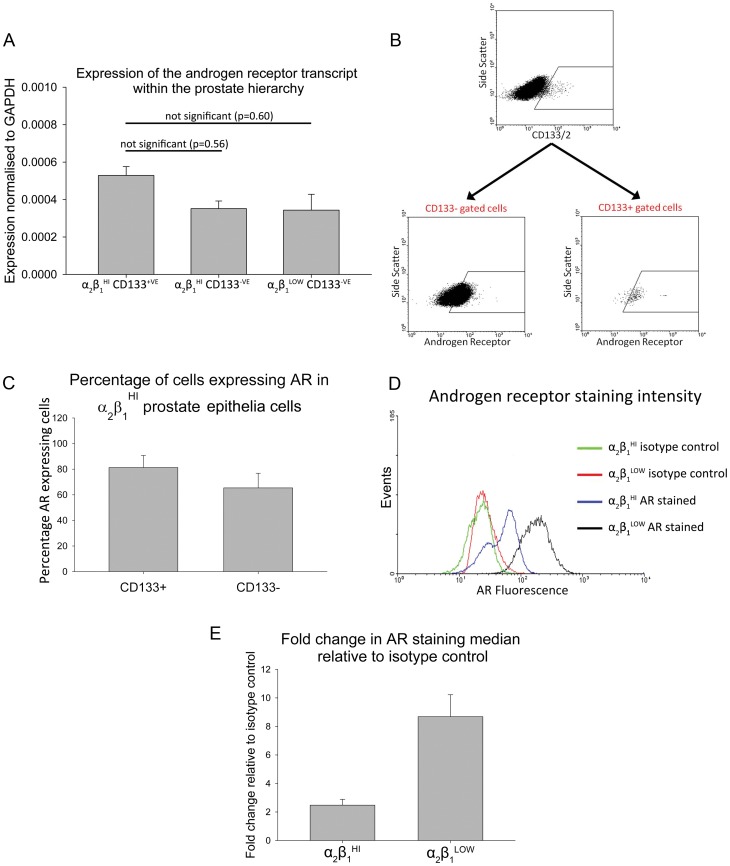
Expression of the AR within the prostate epithelial hierarchy of differentiation. Error bars represent standard error of the mean. **A)** Expression of AR transcript relative to GAPDH. Error bars represent standard error of the mean for n = 10. **B)** Upper dotplot representative of CD133 staining for progenitor α_2_β_1_^HI^ cells. Lower left dotplot representative of AR expression in CD133^–VE^ gated α_2_β_1_^HI^ cells. Lower right dotplot representative of AR expression in CD133^+VE^ gated α_2_β_1_^HI^ cells. Gates were set according to appropriate isotype controls. **C)** Mean percentage of cells expressing the AR in CD133^+VE^ and CD133^–VE^ α_2_β_1_^HI^ cells (n = 6). **D)** Representative histograms for fluorescence of α_2_β_1_^HI^ and α_2_β_1_^LOW^ isotype controls and AR detection. **E)** Mean fold change in median staining relative to isotype control for AR stained α_2_β_1_^HI^ cells and α_2_β_1_^LOW^ cells (n = 6).

### Androgen Receptor was Detected at Low Levels in Prostate Stem Cell Enriched Cells

AR transcript levels were examined in α_2_β_1_^HI^ CD133^+VE^, α_2_β_1_^HI^ CD133^–VE^ and α_2_β_1_^LOW^ CD133^–VE^ cells and were detectable at every stage of differentiation from stem cells through to terminally differentiated cells from each patient sample examined (n = 10) (Figure 3A). Flow cytometry confirmed that similar proportion of CD133^+VE^ stem cell enriched cells and CD133^–VE^ transiently amplifying cells expressed AR protein (Figure 3B), with the mean (±SD) fraction of cells stained positive for AR being 77% (±6%) in α_2_β_1_^HI^ CD133^+VE^ cells and 68% (±12%) in α_2_β_1_^HI^ CD133^–VE^ cells (Figure 3C). Primary progenitor enriched α_2_β_1_^HI^ cells (CD133^+VE^ stem and CD133^–VE^ transiently amplifying cells) demonstrated a clear population shift in fluorescence intensity on the histogram from flow cytometry compared to the isotype control when stained with AR (Figure 3D). In particular, the primary α_2_β_1_^HI^ cells showed 2.54 (SD = ±0.50) fold increase in the median and 2.45 fold (SD = ±0.31) in the peak fluorescence compared to their isotype controls, whereas α_2_β_1_^LOW^ terminally differentiated cells, showed an increase by 8.69 (SD = ±1.54) fold for the median and 6.80 (SD = ±1.11) fold for the peak fluorescence compared to their isotype controls (Figure 3E). In particular, the α_2_β_1_^HI^ progenitor cells did not stain as intensely (3-fold lower) as terminally differentiated prostate epithelial cells (p<0.005) (Figure 3E).

**Figure 4:**
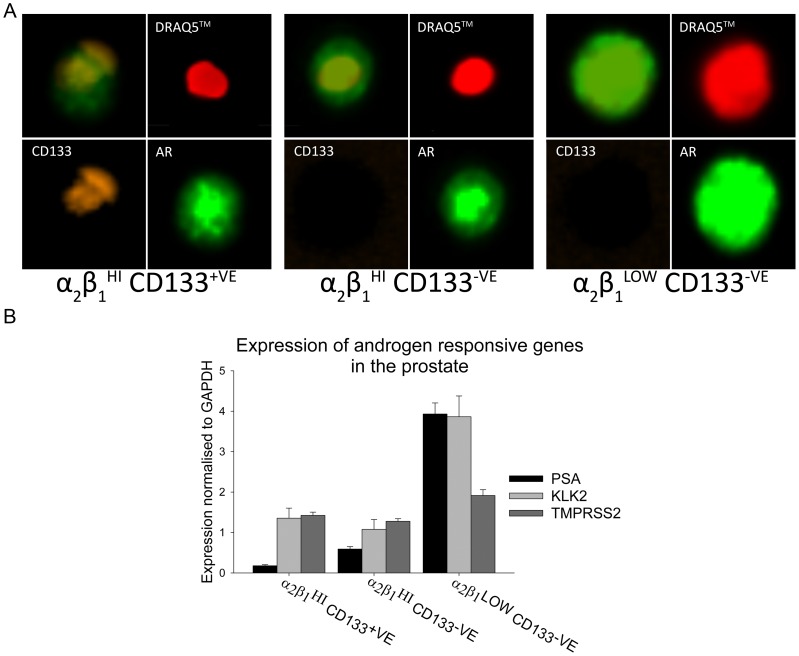
AR expression and activity within the prostate epithelial hierarchy of differentiation. A) Representative images of expression of CD133 and AR counterstained with DRAQ5™ within prostate EpCAM^+VE^ α_2_β_1_^HI^ CD133^+VE^ (Left panel), α_2_β_1_^HI^ CD133^–VE^ (central panel) and α_2_β_1_^LOW^ CD133^–VE^ cells (right panel). **B)** Expression of the AR regulated genes PSA, KLK2 and TMPRSS2 normalised to GAPDH (n = 10) (p<0.001 comparing α_2_β_1_^HI^ and α_2_β_1_^LOW^). Error bars represent standard error of the mean.

To confirm this result, samples were analysed with the ImageStreamX Mark II cytometer, which combines flow cytometery with fluorescent microscopy, allowing visualisation of rare cell events within a sample. AR was once again confirmed to be expressed by α_2_β_1_^HI^ CD133^+VE^ and α_2_β_1_^HI^ CD133^–VE^ cells with higher immunofluorescence expression seen within α_2_β_1_^LOW^ CD133^–VE^ cells (Figure 4A). By counterstaining cells with DRAQ5™, AR localisation could be estimated within the cells in suspension. All three fractions showed AR expression overlaying with the nucleus, suggesting the AR is active throughout prostate differentiation. In order to establish if the AR was functionally active in the rare prostate progenitor cells, expression of androgen regulated genes was examined (Figure 4B). Expression of PSA, KLK2 and TMPRSS2 was confirmed at all stages of prostate differentiation, including activity in the α_2_β_1_^HI^ CD133^+VE^ stem cell enriched cells. Corresponding with the expected increased expression of AR in differentiation, a marked increase in the AR function was demonstrated when comparing the stem fraction to the differentiated fraction: PSA mean fold change = 36.8 (SD = ±15.1), KLK2 mean fold change = 4.3 (SD = ±1.5) and TMPRSS2 mean fold change = 2.3 (SD = ±0.5).

**Figure 5:**
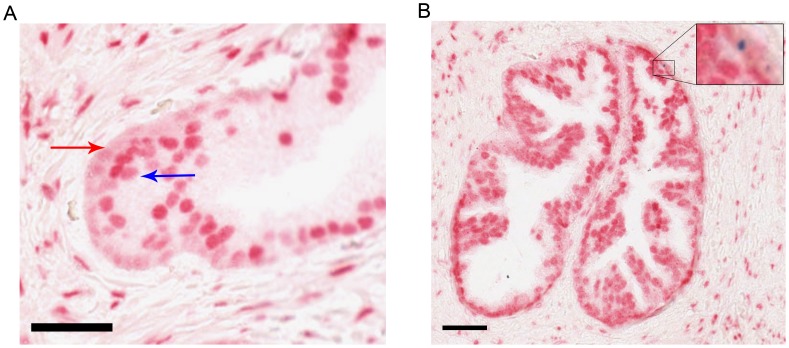
CD133 and AR dual immunohistochemistry. Red stain represents AR expression, blue stain represents CD133 expression, black bars are 50 µm scale bars. A) Representative image of AR staining within prostatic acini. Red arrow identifies basal layer, blue arrow identifies luminal layer. B) Representative image of CD133 and AR expression in the prostate epithelium, insert shows CD133^+VE^ AR ^+VE^ cell within the basal layer.

### Distribution of the Androgen Receptor and Prostate Stem Cells within the Human Prostate Epithelium

To evaluate the expression of AR and CD133 within normal prostate histology, dual alkaline phosphatase staining was employed. Using an optimised AR antibody, it was possible to detect AR within the prostate epithelium in both the luminal layer in addition to low levels within the basal epithelium (Figure 5A). Dual staining also allowed the identification of CD133 positive cells within the prostate epithelium in 2% of sections studied (8/342 sections), with cells always restricted to the basement membrane of the epithelium with localised staining of CD133 in keeping with its expression limited to membrane protrusions with a characteristic punctate pattern [14], [30]. As with the flow cytometry data, the identified CD133^+VE^ cells also expressed AR but at a lower level than that seen in luminal cells within the same gland (Figure 5B).

## Discussion

In this study, we established that AR expression is detectable at low levels in the α_2_β_1_^HI^ CD133^+VE^ stem cell enriched cells of the prostate, using a combination of PCR, dual staining immunohistochemistry and highly specific and sensitive flow cytometry methods. Moreover, we confirmed that AR was active in the stem cell enriched populations by their expression of TMPRSS2, KLK2 and low levels of PSA. However, these expression levels were lower in comparison to differentiated cells.

Previously, α_2_β_1_^HI^ CD133^+VE^ prostate basal stem cells have been shown to lack AR expression [14], [16], and the discrepancies between our data and these studies on CD133^+VE^ and AR expression may be due to the sensitivity and the specificity of the assay used. In particular, we showed that AR levels were 3-fold lower than differentiated cells, which potentially accounts for the difficulties in detecting the AR in extremely rare prostate progenitor cells using less sensitive approaches such as western blot [31]. A recent report identified a highly conserved site in the second intron of the *AR* gene that regulates its expression in response to androgen stimulation and withdrawal [32]. Specifically, it was shown that AR binding to this response element decreased AR gene expression by functioning as a transcriptional suppressor at this site and this may be a mechanism to explain why, despite 3-fold lower levels of AR protein, there were similar levels of AR transcripts in α_2_β_1_^HI^ CD133^+VE^ stem and differentiated cells. Additional explanation for our findings was that in contrast to previous studies we did not culture samples prior to analysis as CD133 expression is altered as a result of *in vitro* conditions [27]. In particular, AR protein undergoes rapid metabolic turnover in prostate cells and *ex vivo* culture rapidly leads to low or undetectable levels of AR protein expression [28], [33]. These issues associated with measuring CD133 and AR may also explain discrepancies between CD133^+VE^ cancer stem cell studies where both the presence and absence of AR is reported [18], [34]. Having validated AR expression in α_2_β_1_^HI^ CD133^+VE^ cells, there is a possibility that there are differential AR functions in prostate stem and differentiated populations [35], particularly given that progenitor cells are also indirectly responsive to androgens through paracrine signalling of growth factors from adjacent AR^+VE^ stroma [28], [36], [37]. Further work exploring this possibility would be of interest and may identify new mechanisms of homeostasis and potential insights into tumourigenesis.

A recent characterisation of prostate stem cells has identified TRA-1-60/CD151/CD166^+VE^ stem cells which were AR^–VE^ [38]. These cells may represent an acquired phenotype following up-regulation of pluripotent markers seen in advanced cancers [39] that drive de-differentiation [40] into a state more in keeping with embryogenesis (e.g. TRA-1-60 expression) and lacking markers of prostate-specific lineage. However, it is accepted that the TRA-1-60/CD151/CD166^+VE^ stem cell may have arisen from a cell of origin for cancer that lacks AR, as in our study we did identify a very small fraction (19%) of α_2_β_1_^HI^ CD133^+VE^ cells lacking AR expression (0.0002% of the total epithelium [14]). This may have clinical relevance where individual tumours may have different cancer stem cell origins, each with their own specific pathobiology requiring tailoring therapies. Further comparative studies are needed to resolve differences between the TRA-1-60/CD151/CD166^+VE^ and the α_2_β_1_^HI^ CD133^+VE^ cancer stem cell models.

In summary, studies of human prostate cancer have demonstrated basal cells, within which the α_2_β_1_^HI^ CD133^+VE^ stem cell resides, are efficient targets of prostate cancer initiation and that AR expression is required [6], [7]. Therefore, the stem cell, which remains the most likely target for transformation, would be expected to have AR expression too. As previously human prostate stem cells were considered to lack AR, the characterisation of AR expression within α_2_β_1_^HI^ CD133^+VE^ cells offers a resolution to a key paradox about the cell of origin in prostate cancer. Further study of specific AR functions in prostate stem and differentiated cells may highlight novel mechanisms of prostate homeostasis and insights into tumourigenesis.

## References

[1] Haffner MC, Aryee MJ, Toubaji A, Esopi DM, Albadine R, et al (2010) Androgen-induced TOP2B-mediated double-strand breaks and prostate cancer gene rearrangements. Nat Genet 42: 668–675. 20601956 10.1038/ng.613 PMC3157086

[2] Lin C, Yang L, Tanasa B, Hutt K, Ju BG, et al (2009) Nuclear receptor-induced chromosomal proximity and DNA breaks underlie specific translocations in cancer. Cell 139: 1069–1083. 19962179 10.1016/j.cell.2009.11.030 PMC2812435

[3] Yu J, Mani RS, Cao Q, Brenner CJ, Cao X, et al (2010) An integrated network of androgen receptor, polycomb, and TMPRSS2-ERG gene fusions in prostate cancer progression. Cancer Cell 17: 443–454. 20478527 10.1016/j.ccr.2010.03.018 PMC2874722

[4] Attard G, Cooper CS, de Bono JS (2009) Steroid hormone receptors in prostate cancer: a hard habit to break? Cancer Cell 16: 458–462. 19962664 10.1016/j.ccr.2009.11.006

[5] Bonkhoff H, Berges R (2010) From pathogenesis to prevention of castration resistant prostate cancer. Prostate 70: 100–112. 19760632 10.1002/pros.21042

[6] Goldstein AS, Huang J, Guo C, Garraway IP, Witte ON (2010) Identification of a cell of origin for human prostate cancer. Science 329: 568–571. 20671189 10.1126/science.1189992 PMC2917982

[7] Lawson DA, Zong Y, Memarzadeh S, Xin L, Huang J, et al (2010) Basal epithelial stem cells are efficient targets for prostate cancer initiation. Proc Natl Acad Sci U S A 107: 2610–2615. 20133806 10.1073/pnas.0913873107 PMC2823887

[8] Owens DM, Watt FM (2003) Contribution of stem cells and differentiated cells to epidermal tumours. Nat Rev Cancer 3: 444–451. 12778134 10.1038/nrc1096

[9] Perez-Losada J, Balmain A (2003) Stem-cell hierarchy in skin cancer. Nat Rev Cancer 3: 434–443. 12778133 10.1038/nrc1095

[10] Leong KG, Wang BE, Johnson L, Gao WQ (2008) Generation of a prostate from a single adult stem cell. Nature 456: 804–808. 18946470 10.1038/nature07427

[11] Wang X, Kruithof-de Julio M, Economides KD, Walker D, Yu H, et al (2009) A luminal epithelial stem cell that is a cell of origin for prostate cancer. Nature 461: 495–500. 19741607 10.1038/nature08361 PMC2800362

[12] Blackwood JK, Williamson SC, Greaves LC, Wilson L, Rigas AC, et al (2011) In situ lineage tracking of human prostatic epithelial stem cell fate reveals a common clonal origin for basal and luminal cells. J Pathol 225: 181–188. 21898876 10.1002/path.2965

[13] Gaisa NT, Graham TA, McDonald SA, Poulsom R, Heidenreich A, et al (2011) Clonal architecture of human prostatic epithelium in benign and malignant conditions. J Pathol 225: 172–180. 21898875 10.1002/path.2959

[14] Richardson GD, Robson CN, Lang SH, Neal DE, Maitland NJ, et al (2004) CD133, a novel marker for human prostatic epithelial stem cells. J Cell Sci 117: 3539–3545. 15226377 10.1242/jcs.01222

[15] Heer R, Collins AT, Robson CN, Shenton BK, Leung HY (2006) KGF suppresses alpha2beta1 integrin function and promotes differentiation of the transient amplifying population in human prostatic epithelium. J Cell Sci 119: 1416–1424. 16554439 10.1242/jcs.02802

[16] Collins AT, Habib FK, Maitland NJ, Neal DE (2001) Identification and isolation of human prostate epithelial stem cells based on alpha(2)beta(1)-integrin expression. J Cell Sci 114: 3865–3872. 11719553 10.1242/jcs.114.21.3865

[17] Litvinov IV, Vander Griend DJ, Xu Y, Antony L, Dalrymple SL, et al (2006) Low-calcium serum-free defined medium selects for growth of normal prostatic epithelial stem cells. Cancer Res 66: 8598–8607. 16951173 10.1158/0008-5472.CAN-06-1228 PMC4124600

[18] Collins AT, Berry PA, Hyde C, Stower MJ, Maitland NJ (2005) Prospective identification of tumorigenic prostate cancer stem cells. Cancer Res 65: 10946–10951. 16322242 10.1158/0008-5472.CAN-05-2018

[19] Mousses S, Wagner U, Chen Y, Kim JW, Bubendorf L, et al (2001) Failure of hormone therapy in prostate cancer involves systematic restoration of androgen responsive genes and activation of rapamycin sensitive signaling. Oncogene 20: 6718–6723. 11709706 10.1038/sj.onc.1204889

[20] Buchanan G, Irvine RA, Coetzee GA, Tilley WD (2001) Contribution of the androgen receptor to prostate cancer predisposition and progression. Cancer Metastasis Rev 20: 207–223. 12085963 10.1023/a:1015531326689

[21] Chen CD, Welsbie DS, Tran C, Baek SH, Chen R, et al (2004) Molecular determinants of resistance to antiandrogen therapy. Nat Med 10: 33–39. 14702632 10.1038/nm972

[22] Kolli S, Lako M, Figueiredo F, Mudhar H, Ahmad S (2008) Loss of corneal epithelial stem cell properties in outgrowths from human limbal explants cultured on intact amniotic membrane. Regen Med 3: 329–342. 18462056 10.2217/17460751.3.3.329

[23] Birgersdotter A, Sandberg R, Ernberg I (2005) Gene expression perturbation in vitro–a growing case for three-dimensional (3D) culture systems. Semin Cancer Biol 15: 405–412. 16055341 10.1016/j.semcancer.2005.06.009

[24] Ross DT, Scherf U, Eisen MB, Perou CM, Rees C, et al (2000) Systematic variation in gene expression patterns in human cancer cell lines. Nat Genet 24: 227–235. 10700174 10.1038/73432

[25] van der Loos CM (2008) Multiple immunoenzyme staining: methods and visualizations for the observation with spectral imaging. J Histochem Cytochem 56: 313–328. 18158282 10.1369/jhc.2007.950170 PMC2326109

[26] Shepherd CJ, Rizzo S, Ledaki I, Davies M, Brewer D, et al (2008) Expression profiling of CD133+ and CD133- epithelial cells from human prostate. Prostate 68: 1007–1024. 18398820 10.1002/pros.20765

[27] Platet N, Liu SY, Atifi ME, Oliver L, Vallette FM, et al (2007) Influence of oxygen tension on CD133 phenotype in human glioma cell cultures. Cancer Lett 258: 286–290. 17977646 10.1016/j.canlet.2007.09.012

[28] Heer R, Robson CN, Shenton BK, Leung HY (2007) The role of androgen in determining differentiation and regulation of androgen receptor expression in the human prostatic epithelium transient amplifying population. J Cell Physiol 212: 572–578. 17541959 10.1002/jcp.21154

[29] Patrawala L, Calhoun-Davis T, Schneider-Broussard R, Tang DG (2007) Hierarchical organization of prostate cancer cells in xenograft tumors: the CD44+alpha2beta1+ cell population is enriched in tumor-initiating cells. Cancer Res 67: 6796–6805. 17638891 10.1158/0008-5472.CAN-07-0490

[30] Corbeil D, Marzesco AM, Wilsch-Brauninger M, Huttner WB (2010) The intriguing links between prominin-1 (CD133), cholesterol-based membrane microdomains, remodeling of apical plasma membrane protrusions, extracellular membrane particles, and (neuro)epithelial cell differentiation. FEBS Lett 584: 1659–1664. 20122930 10.1016/j.febslet.2010.01.050

[31] Bonkhoff H, Remberger K (1993) Widespread distribution of nuclear androgen receptors in the basal cell layer of the normal and hyperplastic human prostate. Virchows Arch A Pathol Anat Histopathol 422: 35–38. 8438556 10.1007/BF01605130

[32] Cai C, He HH, Chen S, Coleman I, Wang H, et al (2011) Androgen receptor gene expression in prostate cancer is directly suppressed by the androgen receptor through recruitment of lysine-specific demethylase 1. Cancer Cell 20: 457–471. 22014572 10.1016/j.ccr.2011.09.001 PMC3225024

[33] Kemppainen JA, Lane MV, Sar M, Wilson EM (1992) Androgen receptor phosphorylation, turnover, nuclear transport, and transcriptional activation. Specificity for steroids and antihormones. J Biol Chem 267: 968–974. 1730684

[34] Vander Griend DJ, Karthaus WL, Dalrymple S, Meeker A, DeMarzo AM, et al (2008) The role of CD133 in normal human prostate stem cells and malignant cancer-initiating cells. Cancer Res 68: 9703–9711. 19047148 10.1158/0008-5472.CAN-08-3084 PMC3072758

[35] Lee SO, Tian J, Huang CK, Ma Z, Lai KP, et al (2012) Suppressor role of androgen receptor in proliferation of prostate basal epithelial and progenitor cells. J Endocrinol 213: 173–182. 22393245 10.1530/JOE-11-0474

[36] Lamb LE, Knudsen BS, Miranti CK (2010) E-cadherin-mediated survival of androgen-receptor-expressing secretory prostate epithelial cells derived from a stratified in vitro differentiation model. J Cell Sci 123: 266–276. 20048343 10.1242/jcs.054502

[37] Cunha GR (2008) Mesenchymal-epithelial interactions: past, present, and future. Differentiation 76: 578–586. 18557761 10.1111/j.1432-0436.2008.00290.x

[38] Rajasekhar VK, Studer L, Gerald W, Socci ND, Scher HI (2011) Tumour-initiating stem-like cells in human prostate cancer exhibit increased NF-kappaB signalling. Nat Commun 2: 162. 21245843 10.1038/ncomms1159 PMC3105310

[39] Bae KM, Su Z, Frye C, McClellan S, Allan RW, et al (2010) Expression of pluripotent stem cell reprogramming factors by prostate tumor initiating cells. J Urol 183: 2045–2053. 20303530 10.1016/j.juro.2009.12.092 PMC4451595

[40] Kumar SM, Liu S, Lu H, Zhang H, Zhang PJ, et al.. (2012) Acquired cancer stem cell phenotypes through Oct4-mediated dedifferentiation. Oncogene. 10.1038/onc.2011.656 PMC3343184 22286766

